# Pathological Anatomy

**Published:** 1879-12

**Authors:** 


*Collaborators:*

DR. H. GRADLE, DR. L. W. CASE, DR. R. PARK,
DR. R. TILLEY.

### PATHOLOGICAL ANATOMY.

FALSE HERMAPHRODISM IN A WOMAN HAVING PASSED HER LIFE AS A MAN.—Drs. Arigo and Fiorani.—(*Revue Méd.*, Sep. 20, 1879, p. 357.)

The patient, named Pagetti, entered the Hospital Maggiore de Lodi, Aug. 12, 1878. Age, 68 years; stature, small; thick set, robust, with tolerably thick gray beard. On entering the hospital he was suddenly taken with a violent fever, with frequent and severe chills, delirium, dyspnœa, cyanosis, coma, and finally death in twenty-four hours.

*Autopsy*—Head has virile aspect, bald on top, with gray hair on the sides and back of neck; nose, aquiline; face, oval; lips, chin, and upper part of neck supplied with somewhat thick beard, of gray color; neck robust; shoulders wide; chest developed; nipples small, without any appearance of mammary glands, as in woman. Abdomen a little prominent, adipose, regular, thighs rounded, legs muscular.

In examining the abdominal viscera, after having pushed back the intestines to get at the bladder, there was discovered, to the extreme surprise of all the assistants, a uterus, with two ovaries and the corresponding ligaments. The examination then became more attentive and minute.

On the exterior, the mons veneris, notably elevated and covered with gray hair in considerable quantity. Lower down, a penis, the size of an ordinary thumb, nearly eight centimeters long, of the color and consistence habitual in old men, and terminated at its free extremity by a well-developed gland of regular form, and proportioned to the size of the penis, but destitute of a urethral meatus. Along the posterior surface, exactly in the site of the urethral canal, between the *corpora cavernosa*, a furrow, not covered with skin, but with a mucous membrane, rendered more consistent from exposure; this furrow extended just to the root of the penis, terminating in an opening of somewhat circular form. By the sides of this opening, and descending towards the perineum, two symmetrical folds, formed like the labia majora, covered externally by scattering hairs, and internally by a membrane more delicate than the skin, and resembling a mucous membrane. Taken as a whole, these folds might be considered as a scrotum, divided by a very strongly marked raphé, and containing atrophied testicles; but, on examining with care, they were clearly seen to be labia majora, and on separating them a membrane was found between, without any orifice, intercepting all communication with the internal parts.

In the interior was found a uterus of the size and form of the virgin uterus, the body and neck being well formed; the broad ligaments, the Fallopian tubes, the ovaries, were in the ordinary condition of virgins, and it was not seen whether or not there existed *corpora lutea*, indices of former ovulation or ovules, and in what state they were.

The bladder having been opened, a bougie was passed through the neck and came out at the opening at the root of the penis. Incision of the vagina, behind the bladder, uncovered the cervix uteri, small, cylindrical, of normal size; another bougie was then introduced into the vaginal canal, which was of the ordinary length and capacity. The bougie passed out of the same orifice as the former one, after having struck against the groove which separated the two elevations forming the labia majora, and being bent a little upward.

The measure of the pelvis was 22 centimeters for the bis-iliac diameter, 9 for the bis-ischiatic, and $10\frac{1}{2}$ for the transverse.

She had all the appearances of a man, but was indeed a bearded woman, without breasts, with a greatly developed clitoris. Nothing was known of her youth, whether menstruation or anything approaching it had ever taken place, or whether her sexual life was that of a man or woman.

